# Invasive Electrophysiology for Circuit Discovery and Study of Comorbid Psychiatric Disorders in Patients With Epilepsy: Challenges, Opportunities, and Novel Technologies

**DOI:** 10.3389/fnhum.2021.702605

**Published:** 2021-07-26

**Authors:** Irena Balzekas, Vladimir Sladky, Petr Nejedly, Benjamin H. Brinkmann, Daniel Crepeau, Filip Mivalt, Nicholas M. Gregg, Tal Pal Attia, Victoria S. Marks, Lydia Wheeler, Tori E. Riccelli, Jeffrey P. Staab, Brian Nils Lundstrom, Kai J. Miller, Jamie Van Gompel, Vaclav Kremen, Paul E. Croarkin, Gregory A. Worrell

**Affiliations:** ^1^Bioelectronics, Neurophysiology, and Engineering Laboratory, Department of Neurology, Mayo Clinic, Rochester, MN, United States; ^2^Biomedical Engineering and Physiology Graduate Program, Mayo Clinic Graduate School of Biomedical Sciences, Rochester, MN, United States; ^3^Mayo Clinic Alix School of Medicine, Rochester, MN, United States; ^4^Mayo Clinic Medical Scientist Training Program, Rochester, MN, United States; ^5^Faculty of Biomedical Engineering, Czech Technical University in Prague, Kladno, Czechia; ^6^The Czech Academy of Sciences, Institute of Scientific Instruments, Brno, Czechia; ^7^Faculty of Electrical Engineering and Communication, Department of Biomedical Engineering, Brno University of Technology, Brno, Czechia; ^8^Department of Psychiatry and Psychology, Mayo Clinic, Rochester, MN, United States; ^9^Department of Otorhinolaryngology, Mayo Clinic, Rochester, MN, United States; ^10^Department of Neurosurgery, Mayo Clinic, Rochester, MN, United States; ^11^Czech Institute of Informatics, Robotics and Cybernetics, Czech Technical University in Prague, Prague, Czechia

**Keywords:** epilepsy, psychiatric disorders, major depression (MDD), SEEG (stereoelectroencephalography), electrocorticography (ECoG), deep brain stimulation, biomarker, neuromodulation

## Abstract

Intracranial electroencephalographic (iEEG) recordings from patients with epilepsy provide distinct opportunities and novel data for the study of co-occurring psychiatric disorders. Comorbid psychiatric disorders are very common in drug-resistant epilepsy and their added complexity warrants careful consideration. In this review, we first discuss psychiatric comorbidities and symptoms in patients with epilepsy. We describe how epilepsy can potentially impact patient presentation and how these factors can be addressed in the experimental designs of studies focused on the electrophysiologic correlates of mood. Second, we review emerging technologies to integrate long-term iEEG recording with dense behavioral tracking in naturalistic environments. Third, we explore questions on how best to address the intersection between epilepsy and psychiatric comorbidities. Advances in ambulatory iEEG and long-term behavioral monitoring technologies will be instrumental in studying the intersection of seizures, epilepsy, psychiatric comorbidities, and their underlying circuitry.

## Introduction

Serious mental illnesses resulting in substantial functional impairment affect over 13 million US adults (Substance Abuse and Mental Health Services Administration, 2020). Neurostimulation-based therapies for psychiatric disorders such as treatment-resistant major depressive disorder (MDD) and obsessive-compulsive disorder (OCD) have garnered considerable research and clinical effort in the past decade (Sullivan et al., 2021). Similar investigations for the treatment of schizophrenia are in an earlier stage of development (Corripio et al., 2020). The neuronal circuitries underlying MDD, OCD, and other psychiatric conditions are incompletely understood. Intracranial electroencephalographic (iEEG) recordings, which measure the local field potential (LFP) of large populations of neurons, are a promising tool for the identification of putative electrophysiologic biomarkers of psychiatric diseases with unique strengths not present in other non-invasive techniques for brain recording in humans (Neumann et al., 2014; Sani et al., 2018; Miller et al., 2019; Veerakumar et al., 2019; Scangos K. et al., 2020; Scangos K. W. et al., 2020; Scangos et al., 2021). The most frequent indication for iEEG recordings with stereoelectroencephalographic (sEEG) electrodes or subdural strip and grid electrodes is seizure onset zone (SOZ) localization as part of presurgical evaluation for drug-resistant focal epilepsy. Given this unique access to invasive recordings, increased effort is being directed to the study of comorbid psychiatric symptoms in people with epilepsy (PWE). This trans-diagnostic approach raises questions about the nature of psychiatric disorders in PWE, confounding factors, and generalizability of findings to patients without epilepsy.

Here, we explore these questions by discussing: (1) psychiatric comorbidities in epilepsy and recommendations for experimental designs; (2) technologies that integrate long-term iEEG with dense behavioral tracking; and (3) lingering questions on how best to study these factors in single and multi-center studies. This review emphasizes both the experimental considerations and opportunities that stem from collaborations with patients who present at the interface of neurology and psychiatry. We hope to convey to the reader that thoughtful approaches to iEEG monitoring, especially long-term ambulatory recordings in naturalistic settings, will help to determine the circuits underlying psychiatric comorbidities in PWE and advance psychiatry research conducted in partnership with PWE.

## Epilepsy and iEEG Monitoring

Epilepsy encompasses a heterogeneous set of disorders with a common presentation of recurrent, spontaneous seizures. A seizure is an abnormal hyper-synchronous or hyperactive brain state, capable of spreading to and recruiting additional brain regions (Fisher et al., 2014). Generalized seizures involve bilateral networks from the onset whereas focal seizures begin in a focal brain region (Fisher et al., 2017). Focal epilepsies are often associated with abnormalities on MRI such as those attributable to gliosis, mesial temporal sclerosis, tumors, or developmental anomalies, though many are not (Carne et al., 2004; Cascino, 2008). Most areas of the brain are potentially implicated in seizure generation or propagation in one type of epilepsy or another, marking considerable potential for overlap with brain regions implicated in psychiatric symptomatology.

Commonly associated with comorbid psychiatric disease, drug-resistant *focal* epilepsy is the primary indication for iEEG monitoring. During invasive monitoring, electrodes are implanted directly on the cortex or into the brain to target deeper regions, with the primary goal of identifying the SOZ. Patients typically spend 4–12 days in the hospital waiting to have their habitual seizures captured on video-EEG (Van Gompel et al., 2008). Seizures encompass a minor proportion of the monitoring period (typically minutes); leaving the patients with considerable time to participate in neuroscience research during their stay, including but not limited to studies of cognition, emotional processing, and electrical stimulation (Lin et al., 2017; Rao et al., 2018; Sani et al., 2018). Surgical resection of the seizure focus, if possible, is the most effective treatment (Cascino, 2004). When the seizure focus cannot be surgically resected, neuromodulatory devices may be implanted to provide therapeutic electrical stimulation (Starnes et al., 2019).

**Figure 1:**
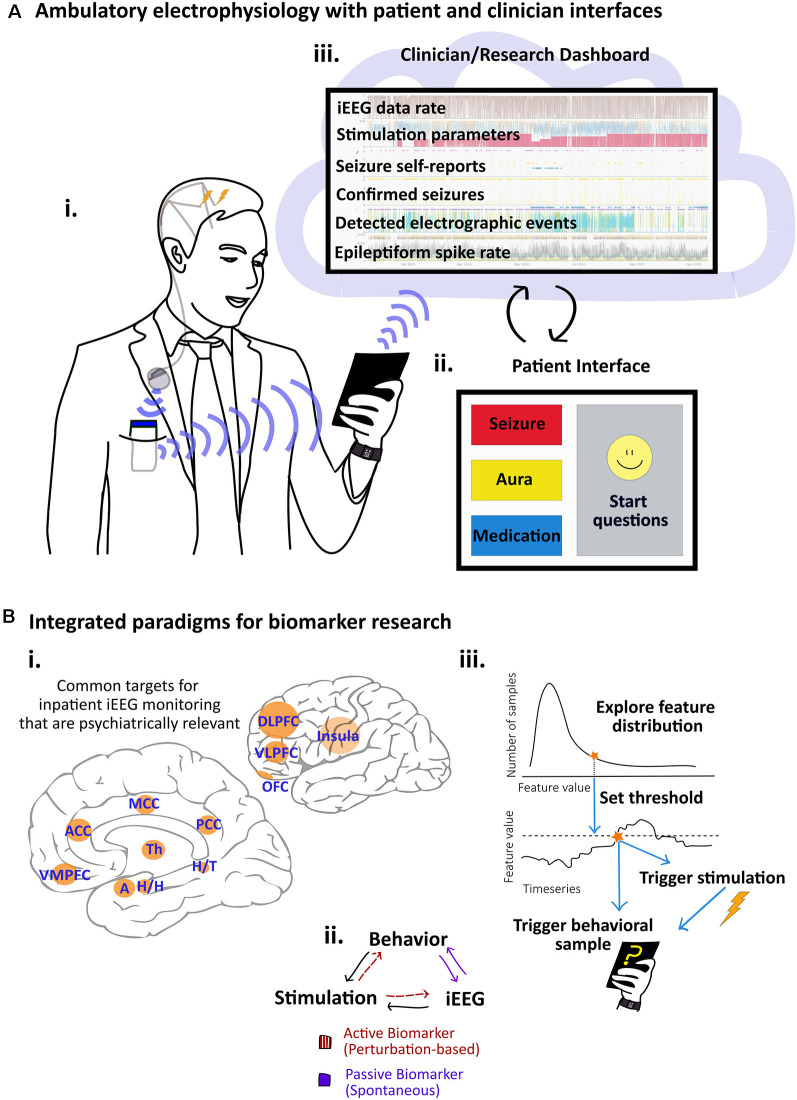
Paradigms and systems for integrated electrophysiology and behavior research. **(A)** System for ambulatory electrophysiology with integrated patient and clinician interfaces. **(i)** Patient with implanted bilateral intracranial depth electrodes connected to subclavicular internal pulse generator (IPG) for deep brain stimulation (DBS) for drug-resistant focal epilepsy. Data from the IPG are transmitted to the pocket-sized relay device which then transmits the data *via* Bluetooth to a small tablet. The patient is shown with a wearable “smartwatch” device to highlight multimodal data options. **(ii)** The patient interface at the tablet is customized to enable the patient to log seizures, auras, and medications, participate in cognitive tasks and ecological momentary assessments (EMAs), and to check system battery levels and data streaming. **(iii)** Data reach the clinical cloud where device status, electrophysiological signals, and patient notes are combined on a clinician dashboard. Custom algorithms run on and off the devices and trends are used to guide remote adjustments of DBS parameters. **(B)** Approaches to biomarker identification. **(i)** The medial and lateral views of the brain show regions implicated in psychiatric pathology that are frequently targeted for Intracranial electroencephalographic (iEEG) recording in patients with epilepsy undergoing invasive monitoring: dorsolateral prefrontal cortex (DLPFC), insula, ventrolateral prefrontal cortex (VLPFC), ventromedial prefrontal cortex (VMPFC), amygdala (A), hippocampal head (H/G) and tail (H/T), thalamus (Th), orbitofrontal cortex (OFC), anterior cingulate cortex (ACC), middle cingulate cortex (MCC), posterior cingulate cortex (PCC). **(ii)** In platforms that integrate stimulation, iEEG, and behavioral assessments, putative biomarkers can be evaluated in active and perturbation-based or more passive and spontaneous approaches. **(iii)** In feature-driven behavioral sampling, we propose using integrated platforms to query behavioral states when ongoing electrographic activity has reached a particular threshold. As opposed to random behavioral queries, this approach may expedite the process of sampling a feature’s full distribution.

Invasive monitoring for the purpose of SOZ localization also involves evaluation of seizure propagation networks, functional mapping, and stimulation trials, all of which simultaneously probe circuits implicated in psychiatric pathology (Kanner and Palac, 2000; Tellez-Zenteno et al., 2007; Kwan et al., 2011; Pham et al., 2017). Common electrode targets include mesial temporal, limbic structures such as the amygdala and hippocampus, as well as the anterior, middle, and posterior cingulate cortex (PCC), orbitofrontal cortex (OFC), insula, frontal cortex, temporal cortex, parietal cortex, dorsolateral prefrontal cortex (DLPFC), and occasionally thalamus (Figure 1B; Yu et al., 2018; Pizarro et al., 2019). Subcortical and cortical targets, including the amygdala, hippocampus, cingulate cortex, and prefrontal cortex are implicated in the circuitry of depression and anxiety (Hare and Duman, 2020).

## Psychiatric Symptoms and Comorbidities in People with Epilepsy

There are several perspectives from which to contextualize psychiatric comorbidities and symptoms in PWE. Although the prevalence of mental health disorders is undoubtedly higher in PWE than in the general population, questions about the causal relationship between epilepsy and psychiatric disease remain unclear, and chicken-egg questions pervade the literature. Is it seizures that provoke psychiatric pathology or psychiatric disorders that increase seizure risk? The longstanding literature on the bidirectional relationship between psychiatric disorders and epilepsy suggests that both are likely true. The temporal dynamics of psychiatric symptoms and their relation to seizures add further complexity. Epilepsy encompasses a heterogeneous group of disorders, warranting special consideration as to which most impact a patient’s risk for psychiatric comorbidity.

Our discussion focuses primarily on MDD and anxiety disorders, excluding personality disorders and other psychiatric diagnoses. The published literature uses a variety of terms to describe mood and anxiety disorders assessed by a variety of methods, including both patient self-reports and clinical diagnostic examinations. In reviewing the literature, we have endeavored to be as specific as possible about psychiatric states, preserving the approach of the primary sources as appropriate.

The approximate lifetime prevalence of any mental health disorder in PWE is 34%, of anxiety-related disorders is 23%, and of MDD is 17%, as compared with 21%, 11%, and 11% in the general population (Tellez-Zenteno et al., 2007). There are many potential explanations for the increased prevalence of psychiatric disorders in PWE. Patient groups with any chronic medical condition, including heart disease, diabetes mellitus, and stroke have an increased prevalence of these same psychiatric disorders (Wells et al., 1988). Although disease burden may explain some of the increased incidences, neurological dysfunction in epilepsy may directly increase the risk for psychiatric comorbidity (Swinkels et al., 2005).

Practically, it can be challenging to determine if psychiatric symptoms are directly associated with pathological electrographic activity in PWE. Subclinical seizures, events with an electrographic correlate that lack the classic behavioral presentation, are common and only detectable with EEG (Zangaladze et al., 2008). Consequently, subclinical seizures are underappreciated outside of hospital settings. Even clinical seizures can be poorly counted by PWE and their caregivers (Cook et al., 2013; Elger and Mormann, 2013). Thus, it is difficult to assert that intermittent behavioral changes are not simply attributable to episodes of interictal epileptiform activity, subclinical seizures, or even seizures that go unwitnessed.

There is an extensive literature on the bidirectional relationship between psychiatric disorders and epilepsy (Mula, 2012a; Kanner et al., 2018). Depression, suicide attempts, and psychiatric hospitalizations are all risk factors for unprovoked seizures (Hesdorffer et al., 2000, 2006; Adelöw et al., 2012). Although some of these findings may be complicated by reporting biases of parents of children with epilepsy, non-epileptic spells misdiagnosed as seizures, and life circumstances such as unemployment and disability (Berg et al., 2017), neurobiological mechanisms support this relationship (Kumar et al., 2007; Kanner, 2008; Kanner et al., 2012; Epps and Weinshenker, 2013; Elkommos and Mula, 2020). For PWE, MDD and mixed mood and anxiety disorders are associated with drug-resistant seizures, increased seizure severity, worse outcomes after epilepsy surgery, and decreased quality of life even if a degree of seizure control is gained after epilepsy surgery (Hamid et al., 2014; Nogueira et al., 2017).

Psychiatric symptoms in PWE are commonly described according to their temporal association with seizures (Kanner, 2009; Berg et al., 2017). Peri-ictal symptoms are directly related to seizures and include pre-ictal symptoms (immediately preceding a seizure), ictal symptoms (during a seizure), and post-ictal symptoms (immediately following a seizure; Swinkels et al., 2005). Inter-ictal symptoms occur in the comparatively long, intervening periods between seizures. The term psychosis of epilepsy is used to capture numerous presentations of inter-ictal and post-ictal psychotic episodes in PWE that differ from primary schizophrenia (Kanner and Rivas-Grajales, 2016). There appears to be a relationship between mood state and impending seizures. Pre-ictal alterations in mood feature prominently in seizure self-prediction by patients with epilepsy along with other common premonitory symptoms such as blurred vision or difficulty concentrating (Willard et al., 2006; Haut et al., 2013). Self-reported stress, lack of sleep, and anxiety also are associated with seizure occurrence (Haut et al., 2007). Inter-ictal symptoms of depression, irritability, anxiety, and euphoria have been grouped under the epilepsy-specific diagnosis of inter-ictal dysphoric disorder (Blumer et al., 2004; Mula, 2013). Although, not all experts concur with these labels as the nosological independence of inter-ictal dysphoric disorder has been questioned (Labudda et al., 2018). Diagnoses of inter-ictal dysphoria, depressive, and anxiety disorders overlap significantly in many patients (Wiglusz et al., 2019).

Psychiatric symptoms in temporal lobe epilepsy (TLE) have been attributed to limbic network pathology, although whether patients with TLE are at higher risk of psychiatric disorders than patients with other types of epilepsy is not clear (Swinkels et al., 2005). Some studies have shown differences in psychiatric disturbances in patients with TLE as opposed to extra-temporal or generalized epilepsies (Perini et al., 1996; Jansen et al., 2019) while others have not (Swinkels et al., 2001). Psychosis of epilepsy, in particular, has been associated with temporal and frontal focal epilepsies (Kanner and Rivas-Grajales, 2016). Additional factors may explain the rates of psychopathology seen in patients with TLE including the presence of multiple seizure types, laterality of the SOZ, and age of onset (Rodin et al., 1976; Hermann et al., 1982). Population level approaches are needed to better untangle these risk factors in patients with TLE specifically.

Psychiatric neuropathology in epilepsy is undoubtedly complex. Despite the many factors that may confound experiments in this area, many of which we will discuss in detail in the following sections, the potential for a deeper understanding of the biology of psychiatric comorbidities of epilepsy is great. Epilepsy is unique in that the electroencephalogram and inter-ictal and ictal iEEG signatures provide biomarkers of the specific circuits involved that can be leveraged to further explore associated psychiatric comorbidities.

## Network Pathologies

Epilepsy, like most psychiatric diseases, is recognized as a network disorder (Kramer and Cash, 2012; Li et al., 2018; Xia et al., 2018). Despite the often focal nature of seizure onset, seizures can have a broad impact on the brain, propagating along and modulating existing circuitry. Chronic seizures are associated with distributed structural and functional changes in cortical and subcortical structures, many of which are involved in psychiatric states and disorders (Bettus et al., 2009, 2010; Bernhardt et al., 2010; Liao et al., 2010; Pereira et al., 2010; Morgan et al., 2011; Kramer and Cash, 2012; Doucet et al., 2013; Maccotta et al., 2013; van Diessen et al., 2013; Keller et al., 2015; Klimeš et al., 2015; Klimes et al., 2016; Tavakol et al., 2019). Volumetric studies suggest partial concordance between structural changes in TLE and MDD namely in the anterior cingulate cortex (ACC), prefrontal cortex, hippocampus, left PCC, and left temporal cortices (Ebmeier et al., 1997; Botteron et al., 2002; Bremner et al., 2002; Videbech and Ravnkilde, 2004; Eker and Gonul, 2010; Kempton et al., 2011; Kanner et al., 2012; Elkommos and Mula, 2020; Schmaal et al., 2020). Larger bilateral amygdala volumes have been reported in patients with TLE and dysthymia than in TLE patients without dysthymia or in healthy controls (Tebartz van Elst et al., 1999). Hippocampal volume loss, a common feature of TLE, can predate symptom onset in MDD and is associated with early presentation and longer symptom duration (McKinnon et al., 2009; Elbejjani et al., 2015; Schmaal et al., 2020). Decreases in functional connectivity within the default mode network have been reported in both patients with MDD (Dichter et al., 2015; Wise et al., 2017) and in TLE patients with depressive symptoms (Chen et al., 2012; Kemmotsu et al., 2013, 2014; Zhu et al., 2018). There may also be concordance between network changes in epilepsy and anxiety disorders, though studies comparing PWE with and without comorbid anxiety are limited (Cendes et al., 1994; Moon et al., 2014, 2015; Moon and Jeong, 2016; Yilmazer-Hanke et al., 2016; Kolesar et al., 2019). Further investigation of the concordance between structural and functional network alterations in various types of epilepsy and psychiatric disorders may reveal core circuit changes that can be expected to yield psychiatric symptoms.

## Electrophysiological Correlates of Psychiatric Symptoms and Disorders from Invasive Recordings

Much of the literature on invasive electrophysiology in patients with psychiatric diagnoses (without epilepsy) has involved iEEG recordings at and near regions established for deep brain stimulation (DBS) treatment of OCD or targeted in clinical trials for MDD. As such, this literature encompasses a mix of different recording sites and putative iEEG biomarkers. Following acute unilateral subcallosal cingulate (SCC) stimulation for MDD, local power changes, notably left-sided theta power increases, were identified as potential guides for stimulation target engagement (Smart et al., 2018). In the bed nucleus of stria terminalis (BNST), patients with MDD showed increased alpha band activity compared to patients with OCD (Neumann et al., 2014). Alpha power pooled across BNST and SCC in MDD correlated significantly with Beck Depression Inventory scores for participants with MDD (Neumann et al., 2014). In characterizing broadband activity and noise in iEEG power spectra, measures of spectral scaling such as *f*^−α^, where α characterizes the slope of a log-log plot of the iEEG power spectrum, were sensitive to phenomena such as task engagement and aging (Pritchard, 1992; Miller et al., 2009; Voytek et al., 2015). An increase in the parameter α in right SCC was associated with treatment response for SCC DBS for MDD (Veerakumar et al., 2019). Subjective improvements of OCD symptoms were associated with increased alpha and beta coherence between the supplementary motor area (SMA) and ventral capsule/ventral striatum (VC/VS) in a patient undergoing VC/VS DBS (Olsen et al., 2020). Additionally, an intraoperative case report in OCD identified a ~35 Hz gamma oscillation in the nucleus accumbens that was modulated by patient obsessions (Miller et al., 2019).

In contrast to the limited range of sites monitored invasively in patients with primary psychiatric disorders, iEEG recordings in PWE cover a broader range of cortical and subcortical areas, often including limbic circuitry. Exploratory investigations of invasive electrographic biomarkers of psychiatric symptomology in PWE have converged on many of the same brain regions implicated in studies of primary MDD and anxiety disorders. Spectral alterations, primarily in the beta band (12–25 Hz) from cortico-limbic iEEG recording sites, including the OFC, cingulate cortex, amygdala, and hippocampus, have been used to distinguish PWE with higher and lower burdens of depressive symptoms (Scangos K. W. et al., 2020). Mood variations have been decoded using spectral-spatial features from limbic, multi-site iEEG networks (Sani et al., 2018). One limbic biomarker, an amygdala-hippocampal connectivity feature in the beta frequency band, was reported to be specific to patients with higher trait anxiety (Kirkby et al., 2018). In PWE with symptoms of depression, electrical stimulation of the lateral OFC was shown to acutely improve mood (Rao et al., 2018), demonstrating the utility of stimulation studies in PWE in the selection of DBS targets to treat primary psychiatric disorders.

These iEEG studies from PWE have laid the groundwork for more recent clinical trials of invasive monitoring in patients with the primary psychiatric diagnosis of MDD, without epilepsy (Scangos K. et al., 2020; Figee and Mayberg, 2021). Scangos et al. (2021) systematically assessed responses to focal stimulation in a patient with severe depression who was implanted with multi-site intracranial electrodes. They revealed an array of emotional responses that were state-dependent and reproducible, demonstrating the promise of patient and circuit-specific approaches to invasive neuromodulation for MDD (Smart et al., 2015).

With the potential for seizure-related and psychiatric comorbidity-related processes to converge in common circuits, increased research into psychiatric processes during iEEG monitoring for PWE is a logical next step. Such intersectional research requires careful consideration of potential confounding factors.

## Rigorous Practices: Confounding Factors and Recommendations for Experimental Designs

In order to successfully investigate psychological processes in PWE, numerous factors must be considered in study design, including the presence of diagnosable psychiatric disorders, severity and duration of psychiatric symptoms, temporal relationship of psychiatric symptoms to epileptic and non-epileptic seizures, presence of additional neurologic and general medical comorbidities, patient demographics, socioeconomic and cultural factors, quality of life, and functional status. Depending on the specific aims of a given investigation, these may be managed through carefully selected inclusion and exclusion criteria and ensuring that study cohorts are large enough to provide adequate power to analyze relevant covariates and confounds. Many of these steps described below are standard practice in iEEG-based neuroscience research (Parvizi and Kastner, 2018; Youngerman et al., 2019). We also propose modifications to behavioral tracking techniques if electrophysiologic data are concurrently available.

### Epilepsy

Variables related to a patient’s seizures must be carefully documented. Most importantly, the temporal association of psychiatric symptoms to seizures should be determined so that peri-ictal and inter-ictal symptoms may be identified accurately. The iEEG time-series must be reviewed for electrographic seizures, and each seizure should denote a two-hour pre-ictal and post-ictal period to be excluded from data analysis (Varatharajah et al., 2017). Clinical seizure prediction algorithms indicate the pre-ictal iEEG changes last for a period averaging around 2 h, although subtle changes in excitability have been detected as far out as 24 h preceding and following seizures (Badawy et al., 2009; Cook et al., 2013). It should be kept in mind that seizures could impact iEEG and MRI at even longer time scales (Cohen-Gadol et al., 2004; Ung et al., 2017). Many patients’ seizures have circadian and multi-day (20–30 day) periodicities (Baud et al., 2018; Karoly et al., 2021). For long-term studies equipped to capture these cycles, it may be necessary to consider the phase of a patient’s seizure periodicities as well. Additionally, functional neurologic (non-epileptic) seizures can occur in patients with epilepsy, highlighting the need to distinguish seizures from behavioral spells when defining ictal and peri-ictal periods (Asadi-Pooya and Sperling, 2015).

Seizure types, semiology, severity, and frequency, as well as the age of onset and presumed etiology, should be noted in supplemental tables together with the location(s) of the SOZ. Signals from SOZ electrodes are traditionally excluded from behavioral analyses, as are those from regions neighboring the SOZ that show an abundance of inter-ictal epileptiform activity. Importantly, epileptiform spiking activity is not specific to the SOZ and can be present on iEEG in brain regions not generating seizures (Lundstrom et al., 2018). Because epileptiform spike rate and amplitude correlate with qualitative seizure probability, studies often elect to exclude electrodes with epileptiform spike rates that exceed a pre-determined threshold (Kucyi et al., 2018; Lundstrom et al., 2018).

Lastly, it is important to note that iEEG is not immune to subtle recorded artifacts, as is commonly assumed (Ball et al., 2009; Kovach et al., 2011; Nejedly et al., 2019a). Concordant electromyography (EMG) and electrooculography (EOG) might be considered in studies where muscle artifacts, including eye movements, might contaminate intracranial signals, especially those evaluating high frequency activity recorded from frontotemporal regions (Jerbi et al., 2009; Worrell et al., 2012).

### Psychiatric Symptomatology and Diagnoses

The full range of psychiatric comorbidity in PWE includes current and lifetime psychiatric disorders as well as peri-ictal and inter-ictal psychiatric symptoms that may not fulfill criteria for specific psychiatric diagnoses. The latter may be transient phenomena associated with seizures themselves or behavioral responses to the challenges of living with epilepsy. For research purposes, the presence or absence of current and lifetime psychiatric diagnoses is best assessed by standardized, structured interviews such as the Mini International Neuropsychiatric Interview or Structured Clinical Interview for the DSM (Sheehan et al., 1998; First et al., 2015). In addition to these categorical measures, interviewer-rated and self-reported assessment tools are available to quantify the severity of psychiatric symptoms as continuous variables. Examples include the Hamilton Depression and Anxiety Rating Scales and Beck Anxiety and Depression Inventories (Hamilton, 1959, 1960; Beck et al., 1961, 1988) as well as comparable scales for other major psychiatric disorders. Epilepsy-specific screening tools such as the Neurological Disorders Depression Inventory for Epilepsy (NDDI-E) are being developed to address the challenge of identifying psychiatric comorbidities in patients taking anti-seizure medications (Gilliam et al., 2006). The different psychological and neurobehavioral constructs guiding each assessment should be considered.

Neurophysiologic signals are dynamic. Their correlation with behavioral manifestations may depend on the time scale and accuracy with which psychiatric symptoms are assessed. Clinical, retrospective self-reports by patients such as the Patient Health Questionnaire 9 (PHQ-9), which quantifies depressive symptoms, show recency effects, and do not represent an “average” of how the patient has felt over a period greater than 1 week (Willard et al., 2006; Aguilera et al., 2015). Ecological momentary assessments (EMA) are a contemporary approach to query patient symptoms in the present moment (Stone and Shiffman, 1994). Regularly implemented EMAs are better at detecting the variability of psychiatric states over time, and at possibly distinguishing patients with more labile symptoms from those with consistently severe presentations (Nahum et al., 2017). Frequent self-reports will be critical to untangle psychiatric symptoms from neurological events. Portable and wearable technologies (e.g., smartphone apps and physical activity monitors) are emerging as effective tools for collecting dense behavioral data on psychiatric states (Insel, 2017).

### Pharmacotherapy

More than 88% of patients taking antiseizure medications (ASMs) experience at least one adverse effect (Baker et al., 1997). The adverse effects of ASMs can involve mood/emotion, cognition, coordination, sleep, weight changes, and cephalgia (Perucca et al., 2009). Psychiatric and behavioral side effects include irritability, anxiety, depressed mood, suicidal ideation, aggression, and psychosis (Perucca et al., 2009). These side effects occur in around 17% of patients on ASMs, with an increased incidence in patients with intractable epilepsy or histories of psychiatric disorders (Chen et al., 2017). Levetiracetam and zonisamide in particular are associated with higher rates of psychiatric and behavioral side effects than other ASMs (White et al., 2003; Chen et al., 2017). Somatic and cognitive symptoms such as weight change, concentration difficulties, appetite changes, sleep disturbance, and fatigue are all associated with ASMs. Although memory difficulties are often attributed to seizure-related neuropathology, memory difficulties can also be attributed to ASM side effects (Mula, 2012b). Many ASMs including gabapentin, valproic acid, carbamazepine, lamotrigine, and topiramate are also used to treat psychiatric conditions including affective, anxiety, and substance use disorders, to name a few (Kaufman, 2011). Thus, disease-related synaptic and circuit-based changes likely exhibit some commonality across epilepsy and psychiatric disorders.

Medications are often tapered during iEEG monitoring and medication documentation is essential to anticipate psychiatric and electrographic side effects. GABA-mediating drugs such as benzodiazepines, are commonly taken for both seizures and anxiety and are associated with significant EEG spectral changes such as decreased alpha and increased beta activity (Buchsbaum et al., 1985). Administration of such medications may not only change EEG spectra but also cause patients to report improvements in self-reported stress and anxiety. Conversely, the withdrawal of therapeutic doses of these medications may produce adverse changes in anxiety or mood states even as the medication confound on the EEG spectra is reduced. The electrographic side effects of ASMs are especially important to consider given that power changes across different frequency bands are emerging as putative indicators of psychiatric pathology and patients with more severe psychiatric comorbidities may be more likely to take these medications (Kirkby et al., 2018; Newson and Thiagarajan, 2019; Scangos K. W. et al., 2020). Deliberate medication tracking is critical to avoid unintentionally labeling the effects of alterations in medication dosing as biomarkers. When possible, keeping stable medication regimens is advantageous. Long-term clinical studies evaluating the efficacy of treatments such as DBS require that patients maintain a stable medication regimen for several months leading up to and throughout the duration of the study (Fisher et al., 2010). For ambulatory iEEG monitoring, medication regimens can be integrated into device platforms to document administration times and doses.

### Non-pharmacologic Therapies

Adjunctive therapies for epilepsy include a variety of non-pharmacologic approaches. Psychological, behavioral, and dietary therapies are particularly relevant due to their potential to alter seizure frequency and presumably influence the networks that generate seizures. Cognitive behavioral therapy and psychotherapy encompass numerous approaches used to manage stress, psychiatric symptoms, and seizures in PWE. Mindfulness, stress management, and exercise regimens including yoga, have shown some promise in reducing seizure frequency and improving quality of life, though larger studies are needed to evaluate the impact on symptoms of MDD (Leeman-Markowski and Schachter, 2017; Panebianco et al., 2017; Noble et al., 2018). Various psychological and behavioral therapies may also help evaluate or measure psychiatrically meaningful characteristics of PWE. Dietary interventions in epilepsy are common. The ketogenic diet has been used widely for pediatric epilepsies and shows some efficacy in adults with epilepsy (Liu et al., 2018). Documentation of these therapies, and others not discussed, is important to understand the range of interventions PWE experience.

### Patient Factors

Patient features such as demographics, cultural factors, quality of life, and functional status, as well as laboratory and imaging data related to neurological and medical comorbidity, are relevant to the interpretability of electrophysiological and behavioral data. Focal seizures can be associated with traumatic brain injury, developmental malformations (both structural and vascular), tumors, gliosis, and mesial temporal sclerosis (Cascino, 2008). Age, gender, ethnicity, socioeconomic status, and quality of life are all meaningful factors when considering a patient’s mental health and functional status (Chen et al., 2018). Substance use constitutes an important factor to record as well, especially in ambulatory studies where participants are no longer in a controlled, inpatient setting. Although large, multi-center studies would be required to explore these as independent factors, these should be reported to determine if the participant sample is particularly enriched with patients that fit a given profile.

### Sleep

Sleep disturbances constitute an important confounding factor when considering psychiatric symptoms such as hyper-, hypo-somnolence, or insomnia in PWE. Sleep difficulties and disorders are common in epilepsy and are likely multifactorial (Grigg-Damberger and Foldvary-Schaefer, 2015; Freeman et al., 2020; Winkelman and Lecea, 2020). In focal, drug-resistant epilepsy, sleep fragmentation is associated with seizures as well as bursts of inter-ictal epileptiform activity (Peter-Derex et al., 2020). Although objective sleep alterations can be attributable to seizures, the relationship between subjective sleep quality and factors such as quality of life and psychiatric comorbidities is more complicated. Self-reported sleep quality, insomnia, and daytime sleepiness in PWE are independently associated with the presence of medical comorbidities and burden of depressive symptoms (Moser et al., 2015; Yang et al., 2016). These factors may interact with medications as well. The tolerability of mood-related side effects from levetiracetam has been associated with patient chronotypes, as patients with morning chronotypes are least likely to tolerate levetiracetam (Taneja et al., 2017). Anti-seizure medications such as clonazepam are directly associated with sleep-related side effects such as daytime sleepiness (Sadler, 1999; Chen et al., 2017).

Sleep disturbance is a core feature of many psychiatric disorders and consequently an important covariate when integrating behavioral metrics in electrophysiological research. Subjective estimates of sleep quality do not always reflect sleep architecture, necessitating both quantitative and qualitative approaches to sleep characterization in integrated electrophysiological and behavioral studies (Armitage et al., 1997; Harvey et al., 2008; Guedes et al., 2016). Portable actigraphs can provide data on sleep times and continuity. Multiple methods for automated sleep staging are available using scalp, subscalp, and intracranial EEG recordings (Gangstad et al., 2019; Kremen et al., 2019; Abou Jaoude et al., 2020). Self-reports such as the Pittsburgh Sleep Quality Index (PSQI) help describe sleep quality with composite quality scores and serve as a screen for potential sleep disorders (Buysse et al., 1989). The effect of sleep quality on the next day’s mood is likely stronger than the reverse, making EMAs important to capture daily fluctuations in perceived sleep quality and their association with psychiatric symptoms (Triantafillou et al., 2019). Accurately tracking sleep, psychiatric symptoms, and seizures in concert will be critical as they constitute three closely related, mutually influencing factors. Lack of sleep is a well-established seizure trigger (Haut et al., 2007). Electrophysiologic markers of cortical excitability, which correlate with seizure risk, have been shown to increase as a function of time spent awake and decrease with ASM use (Meisel et al., 2015), making sleep a non-negligible factor when considering the long-term dynamics of patient behavioral and disease states.

### Electrical Brain Stimulation

The relationship between electrical brain stimulation (EBS) for epilepsy and mood remains unclear, despite its clear clinical importance. Current evidence suggests that therapeutic EBS for epilepsy does not adversely impact mood or cognition (Chan et al., 2018). For the anterior nucleus of the thalamus (ANT) DBS, reversible, parameter-dependent side effects including anxiety and nocturnal arousals have been reported (Fisher et al., 2010; Voges et al., 2015; Järvenpää et al., 2018). Long-term efficacy and safety evaluation of ANT DBS identified only three-device related depression events of 90 participants at 5-year follow-up (Salanova et al., 2015). Most patients showed improvements in anxiety, attention, and executive function compared to baseline. Though, outside of that clinical trial, there has been a reported case of persistent psychiatric side effects during ANT DBS and following discontinuation of ANT DBS (Doležalová et al., 2019). For patients with intractable epilepsy receiving vagus nerve stimulation (VNS), the impact on mood appears to be favorable; VNS is an FDA-approved therapy for treatment-resistant depression (Aaronson et al., 2017; Chan et al., 2018; Elger et al., 2000). Data describing mood changes with Responsive Neurostimulation (RNS) are limited, but some studies have reported moderate improvements in BDI-II scores (Meador et al., 2015; Aaronson et al., 2017). The high prevalence of psychiatric comorbidities in PWE, in general, can complicate the interpretability of psychiatric side effects during therapeutic stimulation. Studies with concurrent invasive stimulation should document and report stimulation periods, parameters, brain targets, and durations. Again, the time scales over which ESB can affect brain circuits are not well understood and careful attention should be paid to their potential impact on experiments.

### Paradigms to Discover Electrophysiologic Signatures of Psychiatric Symptoms and Diagnoses

The fundamental question of what constitutes biomarkers of psychiatric disorders remains unresolved (Ewen et al., 2021). If each of the major psychiatric disorders arises from a unique disturbance of one or more closely related brain processes, then it may be possible to discover disease-specific biomarkers for each one. If, however, the major psychiatric disorders arise from a coalescence of disturbances in fundamental brain processes, simultaneously or sequentially, then biomarkers are more likely to exist for transdiagnostic changes that may co-occur in unique patterns for each major group of psychiatric disorders. The latter concept is captured by the Research Domain Criteria (RDoC) Initiative at the National Institute of Mental Health, which emphasizes the investigation of broader, transdiagnostic, psychological, and biological processes (e.g., mood dysregulation) that may underlie clusters of symptoms that are components of more than one categorical disorder (Thomas Insel et al., 2010; Widge et al., 2017; Ahmed et al., 2018). Research evaluating psychiatric symptomatology with iEEG will have to progress together with research that identifies, defines, deconstructs, and validates brain processes related to core psychiatric phenomena and their putative psychiatric biomarkers (Ewen et al., 2021), along with their inter-individual variation. By directly recording from many of the circuits implicated in RDoC domains, future research with iEEG is positioned to inform and advance these neurobehavioral constructs.

The behavioral context of a brain signal lends complexity to the search for biomarkers as well. Feature space, electrographically and behaviorally, is very large. The brain occupies various states of arousal, wakefulness, and sleep that can be altered in neurological and psychiatric disease (Pfaff et al., 2008; Koch et al., 2016). Patient behavioral states and responses can be provoked as in obsessions in OCD or with the presentation of emotionally evocative images in a task (Kragel and LaBar, 2016; Miller et al., 2019). Acute fluctuations in symptom severity are overlaid on chronic symptom burden (Starr and Davila, 2012). The significance of different electrophysiologic features may vary with time scale as well. In response to stimulation, acute changes could be attributable to transient circuit modulation whereas chronic changes might capture long-term plasticity (Herrington et al., 2016). Responses to stimulation vary by brain region and electrode placement (Basu et al., 2019). Continued efforts to characterize and quantify psychiatric disorders and their development across lifespans and heterogeneous patient groups will be instrumental in guiding iEEG-based approaches to psychiatric symptomatology. Importantly, inpatient iEEG recordings, with multi-site electrode placement, especially in psychiatrically relevant brain regions (Figure 1B), will continue to serve as an important source for preliminary biomarker discovery and evaluation.

Emerging platforms for chronic invasive, ambulatory electrophysiology will offer solutions to some of the above challenges, especially with regards to types, time scales, and state-dependence of putative iEEG biomarkers. Behavioral sampling paradigms will have a profound influence on the type, dynamics, and reliability of putative iEEG biomarkers. To illustrate this concept, we have diagrammed two approaches to defining biomarkers in the contexts of stimulation and behavioral sampling (Figure 1B). Active, stimulation-based approaches involve perturbation-based biomarkers such as evoked potentials or stimulation-driven behavioral changes, concurrently evaluated with iEEG (Figure 1Bii). Passive approaches, cognizant of a potential iEEG biomarker’s variability and statistical properties (Figure 1Bii), can leverage ambulatory iEEG recording for feature-driven behavioral sampling (Figure 1Biii). When an iEEG feature is detected as representing a given region of the biomarker’s distribution, an integrated system could record additional physiologic data streams that may covary (e.g., heart rate variability) and prompt patients to provide behavioral assessments. The full range of the continuous feature would then be represented by a range of co-varying physiological variables and behavioral samples. Unlike random sampling, which is more akin to current approaches (Sani et al., 2018), this approach would enable exploration of the tails of an electrophysiologic marker’s distribution, capturing electrophysiologic extremes that could carry behavioral significance. For example, a device-detected seizure could trigger a recording of physiological data from a wearable device and a simultaneous cognitive assessment on a smartphone. Smarter systems for ambulatory iEEG recording will enable more elegant experimental designs and sampling paradigms, hopefully decreasing the patient burden and improving reproducibility.

## Clinical Technologies for Ambulatory, Invasive Electrophysiology

Numerous opportunities now exist to integrate invasive (implantable) and noninvasive (portable or wearable) devices for monitoring outside of the clinic. With the primary goal of improving therapy for epilepsy, tools to deliver stimulation and predict and detect seizures are becoming increasingly sophisticated, moving toward a paradigm where patients are empowered to customize their disease management. Here, we describe implantable devices used to treat drug-resistant epilepsy that have both iEEG “sensing” and “recording” capabilities. Such invasive neuromodulatory EBS devices are increasingly capable of analyzing neural activity.

The Neuropace Responsive Neurostimulation RNS ^®^ device is an FDA-approved neurostimulator designed to detect and interrupt seizure activity by delivering stimulation to the putative SOZ (Geller, 2018). The RNS continuously senses iEEG. It provides counts of detected inter-ictal epileptiform abnormalities (epileptiform activity or seizures), but can only store a limited duration (6 min) of recorded LFP data that can be uploaded to the company’s cloud database for viewing (Duun-Henriksen et al., 2020). An ongoing clinical trial is leveraging the RNS system for personalized, closed-loop neurostimulation for treatment-resistant depression (NCT04004169). Clinical investigations of seizure periodicities using RNS data have highlighted the potential to untangle associations between patient states and electrographic events in long-term studies (Walker et al., 2020).

The Medtronic Percept^TM^ PC Neurostimulator is an invasive neurostimulator with FDA approval to treat Parkinson’s disease, essential tremor, and epilepsy, and is under a humanitarian device exemption for OCD and dystonia. The device has several recording modes. In the outpatient setting, the clinician can quickly survey power spectral data in 30-s epochs (one per hemisphere). The clinician can predefine a central frequency ranging from 2 to 95 Hz with a 5 Hz bandwidth, which is then saved as averaged power in band values from 10-min epochs to be continuously tracked outside of the clinic (Goyal et al., 2021). These capabilities enable further long-term tracking of established and emerging biomarkers.

The Medtronic Summit RC+S^TM^ device is a rechargeable, sensing neurostimulator currently used in research applications under an FDA investigational device exemption. It can deliver electrical stimulation and has four dynamically selectable (from the total 16 contacts) bipolar sensing channel pairs. Bipolar LFP data from the four channel pairs are continuously transmitted to a relay device and onto a tablet (Figure 1A). The Summit RC+S^TM^ and disease-specific, customized platforms are currently being deployed to study Parkinson’s disease, MDD, OCD, and epilepsy (Clinical trials NCT03582891, NCT04106466, NCT03457675, NCT03946618). Although the RC+S device remains limited to research use, it provides an exciting window to the future of integrated electrophysiological and behavioral studies.

Regarding integrated systems, clinically available infrastructures to support sensing and closed-loop responsive stimulation devices lack the computing power and data storage capacity needed to track the electrophysiologic, therapeutic, and behavioral features associated with seizures. The Mayo Epilepsy Personal Assistant Device (EPAD) is a neurological disease management system that integrates the implanted device, intracranial EEG telemetry, electrical stimulation, behavioral state classifiers, remote parameter control, hand-held computational device, and cloud environment (Kremen et al., 2018; Sladky et al., 2021). Our group has developed custom software that runs on the patients’ tablet computer and enables continuous streaming of LFP data to a physician cloud; allowing remote adjustment of stimulation parameters and customized algorithms (Kremen et al., 2018). We designed this firmware/software ecosystem as a “neural coprocessor” to create a bi-directional neural interface for patient and clinician use. The system is an advance toward modular, integrated systems for neural prostheses that can be configurable to patient needs and disease-specific pathology (Stanslaski et al., 2018). Machine learning techniques for spike and seizure detection are incorporated into the system as well (Nejedly et al., 2019b). Figure 1A provides a high-level description of the system capabilities, emphasizing their relevance to behavioral integration.

With this framework, multiple streams of data are collected from the Summit RC+S^TM^ device. In terms of sensing, LFP timeseries data are continuously recorded from the four bipolar electrode pairs with an adjustable sampling rate of 250 or 500 Hz and a customizable selection of electrode pairs including stimulating and sensing contacts. Stimulation parameters (frequency, amplitude, and pulse width) are remotely programmable and stored. The EPAD tablet allows for patient annotation of medication administration, auras, and seizures, as well as cognitive tasks and self-reported mood queries for long-term tracking of cognitive performance and psychiatric symptoms.

Implanted neuromodulation devices are evolving rapidly, with increasing capacity for data sensing, adaptive stimulation, and closed-loop applications. Table 1 highlights some critical domains for untangling psychiatric features and chronic seizures and the limitations of current clinical hardware in addressing these domains. Novel, research-grade devices are beginning to fill these gaps. Brain computer interfaces for patients with tetraplegia, for example, have also fostered advances in implanted technologies for chronic brain recordings (Simeral et al., 2021). Multifunctional research platforms that integrate these emerging devices with wireless stimulation control, peripheral biosensors, and environmental features as in virtual reality should further guide and inform the next generation of clinical hardware (Topalovic et al., 2020).

**Table 1:** Quantifying factors that contribute to psychiatric symptoms in people with epilepsy: approaches and devices.

Potential confounding factors	Methodological recommendations to quantify potential confounding factors	Necessary device specifications			
		*Sensing*	*Recording*	*Detection*	*Integrated Annotation*
Seizures	Identify and annotate seizures, patient reported semiology, and electrographic characteristics.	✓	✓	✓	✓
Inter-ictal epileptiform activity	Identify and quantify changes in epileptiform spike rate.	✓	✓	✓	
Medications	Document administration times and doses.				✓
Psychiatric symptoms and comorbidities	Track ecological momentary assessments and retrospective self-reports.				✓
Sleep	Track self-reported sleep quality and objective sleep architecture.	✓	✓		✓
Electrical brain stimulation	Track stimulation parameters and remotely adjust stimulation paradigms.				✓
Sensing devices	Clinical device applications	Current device specifications			
Neuropace RNS^®^	FDA approved for drug resistant focal epilepsy.	✓	✓ Stores 6 min of scheduled or event-triggered LFP (iEEG)	✓ Embedded detector with programmable tuning	✓ Patient event annotations (magnet)
Medtronic Percept^TM^ PC	FDA approved for epilepsy, Parkinson’s disease, essential tremor; Humanitarian device exemption for obsessive compulsive disorder and dystonia.	✓	✓ Stores 10 min average of selectable PIB	✓ Embedded detector based on 10-min PIB	
Medtronic Summit RC+S^TM^	FDA investigational device exemption (can be integrated with Mayo EPAD)	✓	✓ Continuous telemetry of LFP (iEEG) data	✓ Embedded and off-device detectors	✓ Integrated annotations and EMA

*Major confounding factors in the study of psychiatric symptoms in people with epilepsy (PWE) and relevant experimental considerations and device capabilities are described here. Devices with “sensing” capabilities can perceive or handle data, whereas those with “recording” capabilities can store or stream data. The Neuropace RNS^®^ device, for example, continuously senses and processes data with an adjustable embedded detector but can only store up to 6 min of raw data at any given time, in addition to the event detection log. Note: although there are other invasive brain stimulation devices with FDA approval to treat epilepsy, this table focuses on invasive brain stimulation devices that have sensing capabilities. Abbreviations: PIB, power in band; EMA, ecological momentary assessment; LFP, local field potential; iEEG, intracranial electroencephalography*.

## Discussion

PWE have been critical partners in invasive neurophysiology research since the beginnings of epilepsy surgery (Penfield and Boldrey, 1937). The concern about the generalizability of psychiatry research with PWE is valid but should not distract from the genuine need for better understanding and management of psychiatric comorbidities in epilepsy and neurological disease in general. The burden of psychiatric symptoms has a greater impact on QOL than seizure control and psychiatric comorbidities are associated with treatment resistance in epilepsy. There is a critical need for partnership with psychiatry to better care for PWE (Kanner, 2016; Fasano and Kanner, 2019). PWE especially stand to benefit greatly from increased attention paid to psychiatric comorbidities and symptomatology in epilepsy research and invasive monitoring.

### Experimental Designs: Individuals and Populations

A practical question that emerges from this discussion of confounding factors in intersectional studies concerns the study design that is best suited to manage variability. High-resolution studies using intensive, chronic, ambulatory collection of both iEEG and behavioral data would yield more personalized findings. Large, multi-center trials, may capture more variability between participants. The fact that placement of iEEG electrodes is clinically dictated and patient-specific presents another challenge for reliable biomarker identification. Fortunately, there is considerable consistency of iEEG electrode targeting for inpatient iEEG monitoring, especially for TLE. The same recording sites are typically used across subjects, be they in the left or right hemisphere. Given the frequency with which these iEEG recordings are performed at tertiary medical centers, it is realistic to anticipate that such multicenter trials could enroll an adequate number of participants over time. Standardization for DBS clinical trials and iEEG research across multiple centers has been successfully demonstrated (Kerrigan et al., 2004). The DARPA Restoring Active Memory (RAM) study is a powerful example of how pooling iEEG data from multiple centers performing the same experiments can expedite and strengthen important discoveries in neuroscience (Kucewicz et al., 2018).

### Intersectional Approaches to Circuit Discovery

It is not yet known to what extent biomarkers of psychiatric symptoms discovered in PWE would be generalizable to the patients suffering from primary psychiatric disorders. At a minimum, data obtained from PWE using systems such as the ones described in this report will drive further advances in technologies and techniques that will increasingly become available for investigating and treating patients with primary major psychiatric illnesses. Of course, each technology has its own set of limitations including safety, tolerability, signal integrity, user acceptability, and required technological literacy, privacy, and access (Zuk et al., 2018). Such integrated and ambulatory approaches may prove especially useful in applications such as MDD or Alzheimer’s disease where trials for DBS therapies have shown delayed or limited efficacy (Holtzheimer et al., 2017; Leoutsakos et al., 2018; Crowell et al., 2019). The convergence of data obtained from patients with neurologic and psychiatric illnesses, including PWE and others, promises to unlock greater insights into fundamental brain processes underlying human neuropsychiatric disease. Two of the neurologic disorders for which implantable devices are now being used (epilepsy and Parkinson disease) have high rates of psychiatric morbidity (mood, anxiety, cognitive, and psychotic symptoms) and the psychiatric disorder most commonly treated with implantable devices (OCD) has a high rate of neurologic comorbidity (motor and vocal tics; Gomes de Alvarenga et al., 2012). These illnesses, however, are just a starting point. The emergence of user-friendly, implantable, wearable, and portable technologies marks new avenues to explore the multiscale dynamics underlying human brain states and drive developments of adaptive neuromodulatory therapies (Provenza et al., 2019).

## Conclusion

The presence of psychiatric comorbidities and symptoms in PWE, the most extensively electrographically monitored patient population, constitutes a critical opportunity to explore brain networks in psychiatric disorders. Careful consideration of confounding factors in experimental designs, together with the next generation of ambulatory, iEEG monitoring technologies, promises to help characterize the circuitry driving psychiatric comorbidities in PWE and possibly patients with primary psychiatric disorders. We hope that continued prioritization of such intersectional and translational research will strengthen the ties between the fields of neurology and psychiatry, benefitting the many patients who lie at their interface.

## Data Availability Statement

The original contributions presented in the study are included in the article, further inquiries can be directed to the corresponding author.

## Author Contributions

IB and GW conceived the presented idea. IB outlined and wrote the article with GW and PC with additional subsection contributions as described here. VS, PN, BB, DC, FM, TP, VK, VM, and LW contributed to the discussion of novel technologies and preliminary results. NG, BL, GW, JS, and PC contributed to the background regarding epilepsy and psychiatric comorbidities and clinical overview of confounding factors. JG and KM contributed to the discussion of surgery, emerging electrophysiologic biomarkers, and figure design. All authors contributed to the article and approved the submitted version.

## Conflict of Interest

PC has received research grant support from Neuronetics, Inc., NeoSync, Inc., and Pfizer, Inc. He has received grant in-kind (equipment support for research studies) from Neuronetics, Inc. and MagVenture, Inc. He has served as a consultant for Myriad Neuroscience and Procter & Gamble. BL, BB, and GW are named inventors for intellectual property developed at Mayo Clinic and licensed to Cadence Neuroscience Inc. BL has waived contractual rights to royalties. GW has licensed intellectual property developed at Mayo Clinic to NeuroOne, Inc. GW and BL are investigators for the Medtronic Deep Brain Stimulation Therapy for Epilepsy Post-Approval Study (EPAS). BL, BB, and GW are investigators for Mayo Clinic and Medtronic NIH Public Private Partnership (UH3-NS95495). GW assisted in a Mayo Clinic Medtronic sponsored FDA-IDE for the investigational Medtronic Activa PC+S device. VM discloses that she has received compensation from an internship with Medtronic, Inc., for work unrelated to the current publication. IB has received compensation from an internship with Cadence Neuroscience Inc., for work unrelated to the current publication. JS has received compensation from Inno.health for work unrelated to the current publication. The remaining authors declare that the research was conducted in the absence of any commercial or financial relationships that could be construed as a potential conflict of interest.

## Publisher’s Note

All claims expressed in this article are solely those of the authors and do not necessarily represent those of their affiliated organizations, or those of the publisher, the editors and the reviewers. Any product that may be evaluated in this article, or claim that may be made by its manufacturer, is not guaranteed or endorsed by the publisher.
